# Magnetic Resonance Imaging of the Living Brain: Evidence for Brain Degeneration Among Alcoholics and Recovery With Abstinence

**Published:** 2008

**Authors:** Margaret J. Rosenbloom, Adolf Pfefferbaum

**Keywords:** Alcoholism, brain, brain function, brain structure, neuropathology, cognitive impairment, brain imaging, neuroimaging, magnetic resonance imaging (MRI), functional magnetic resonance imaging (fMRI), diffusion tensor imaging (DTI), in vivo imaging studies, frontal cortex, white matter, human studies, animal models, longitudinal studies

## Abstract

Magnetic resonance imaging (MRI) provides a safe, noninvasive method to examine the brain’s macrostructure, microstructure, and some aspects of how the living brain functions. MRI is capable of detecting abnormalities that can occur with alcoholism as well as changes that can occur with sobriety and relapse. The brain pathology associated with chronic excessive alcohol consumption is well documented with imaging of the living body (i.e., in vivo imaging). Consistent findings include shrinkage of the frontal cortex,^1^ underlying white matter, and cerebellum and expansion of the ventricles. Some of these changes are reversible with abstinence, but some appear to be enduring. Research showing correlations between brain structure and quantitative neuropsychological testing demonstrates the functional consequences of the pathology. In addition, functional imaging studies provide evidence that the brain compensates for cognitive deficits. The myriad concomitants of alcoholism, the antecedents, and the consumption patterns each may influence the observed brain changes associated with alcoholism, which tend to be more deleterious with increasing age. The multifaceted nature of alcoholism presents unique challenges and opportunities to understand the mechanisms underlying alcoholism-induced neuropathology and its recovery. Longitudinal MRI studies of animal models of alcoholism, however, can address questions about the development and course of alcohol dependence and the scope and limits of in vivo degeneration and recovery of brain structure and concomitant function that may not be readily addressed in clinical studies.

Alcohol use disorders are characterized by the excessive consumption of alcohol despite its interference with an individual’s physical, mental, interpersonal, and social well-being. These harmful behavioral effects are mediated through the brain, which can undergo changes in structure, function, and basic physiology. Some studies (e.g., Cardenas et al. 2007; Gazdzinski et al. 2005*a*; Pfefferbaum et al. 1995) have shown evidence for recovery with extended sobriety, but some of the brain changes may persist even after extended sobriety, reflecting diminished ability to maintain function when confronted by degenerative processes (i.e., functional reserve) and decreased ability of the brain to change (i.e., plasticity). These persistent alcohol-induced brain changes themselves then may contribute to the self-sustaining nature of alcoholism.

This article reviews studies using three different types of magnetic resonance imaging (MRI)^2^ brain scanning to measure the effects of excessive chronic alcohol consumption on brain size or shape (i.e., macrostructure), tissue quality (i.e., microstructure), and function (i.e., localized blood flow in support of cognitive or motor tasks). To assess the immediate effects of chronic excessive drinking on the brain and cognitive and motor performance, investigators most commonly test alcoholics shortly after they enter treatment and compare them with low-alcohol–consuming study participants (i.e., control subjects) of similar age, sex, and socioeconomic level.

To test whether the effects of excessive alcohol consumption persist after sobriety is maintained, investigators may compare alcoholics with different lengths of sobriety or preferably follow the same people over time and retest them after varying periods of sobriety. Importantly, these longitudinal studies also require retesting a comparison group of low-alcohol drinkers to control for normal changes in aging and distortion inherent to MRI (i.e., scanner drift) over time. Tests to measure the extent and time-course of such recovery in humans typically are initiated while patients are in alcohol treatment. Such tests track the effects of withdrawal and short-term sobriety. Follow-up studies require tracking patients after discharge, when they have moved back into the community and either maintained sobriety or relapsed into drinking. Longer-term studies therefore take the form of naturalistic rather than controlled experiments because the investigator has no control over whether patients will maintain sobriety or resume drinking and what level of drinking will be embraced. Furthermore, some participants will drop out of the study, affecting the representativeness and size of the followup sample.

Even cross-sectional comparisons of problem drinkers with light drinkers must consider that many factors in addition to alcohol consumption may differentiate the groups. Many alcoholic patients also have comorbidities that can affect the brain, including mood disorders, abuse of other substances, and infection from the hepatitis C virus. Thus, investigators performing these studies ideally must screen study participants for these other illnesses and conditions. Furthermore, people with alcoholism may suffer from occult liver disease, malnutrition, and head trauma and are more likely to be chronic smokers than people who are not dependent on alcohol. Each of these factors has its own consequence on the brain (Brody et al. 2004; Gallinat et al. 2006; Grover et al. 2006). Samples of chronic alcoholics also tend to differ from the comparison group of low-alcohol drinkers on variables such as socioeconomic status, mental status prior to onset of alcoholism, and family history of alcoholism (Tarter and Edwards 1986). The amount of alcohol consumed over a lifetime, the pattern of drinking—whether regular or sporadic—and the frequency and intensity of withdrawals also may contribute to how alcohol affects the brain (Anstey et al. 2006; Bjork et al. 2003; Pfefferbaum et al. 1988; Sullivan et al. 1996).

**Structural Magnetic Resonance Imaging**

Conventional structural magnetic resonance imaging (MRI) takes advantage of the fact that different tissue types in the brain contain different proportions of water, which influences their MRI-visible signal (see figure 1A). Gray matter is about 80 percent water and consists of nerve cells (i.e., neurons) and glial cells, which support neurons. White matter is about 70 percent water and consists of long fibers called axons that carry information between neurons. Cerebrospinal fluid (CSF) fills the spaces between the infoldings of the brain, the ventricular system in the brain, and the space surrounding the brain within the skull and is about 100 percent water. White matter is paler in color than gray matter because the axons are enwrapped in myelin, which is a system of cell bodies (i.e., oligodendrocytes) that wind around the axon and augment neural transmission. The axons form fiber tracts linking nearby and distant neurons across different brain regions (i.e., white matter tracts) (see figure 2). With structural MRI, researchers can identify differences in brain tissue types and structures by manipulating the way in which water protons are excited, yielding intensity differences between tissue types that allow researchers to map gross brain neuroanatomy (i.e., macrostructure). Intensity differences also are used to differentiate gray matter, white matter, and CSF. Volumes of these tissue types can then be measured in different regions of the brain. In addition, specific neuroanatomic structures, such as the corpus callosum, hippocampus, and basal ganglia, can be outlined and their volumes measured. MRI is a safe, noninvasive method to examine the structure of living humans and animals and is powerful enough to detect changes in brain structure that can occur with alcohol sobriety (see figure 5).

Most brain-imaging research focuses on alcohol-dependent individuals recruited through treatment programs. However, the majority of people who meet the criteria for alcohol dependence never seek treatment for their condition (Cohen et al. 2007). Some treatment-naïve alcoholics also show brain alterations (Fein et al. 2002; Gazdzinski et al. 2008*a*), but their lifetime trajectory of alcohol use differs from treatment seekers (Fein and Landman 2005), suggesting yet another dimension of variability to be considered when designing studies of the effect of excessive alcohol consumption on the brain. Despite these challenges in conducting in vivo imaging studies of the consequences, studies generally are in agreement over the broad pattern of disruption observed and find that observations made in the living brain with MRI are consistent with a large literature of pathological data obtained by examining brains postmortem.

The following sections examine MRI evidence for brain abnormalities on both macrostructural and microstructural levels (using conventional MRI and diffusion tensor imaging^3^ [DTI], respectively). Additional studies review the efficiency with which blood flow serves the activation of nerve cells (i.e., neurons) called upon when people perform experimental cognitive tasks (i.e., functional MRI [fMRI], which is described in the textbox on page 370). Cross-sectional studies (reviewed below) of the effects of excessive alcohol consumption on the brain conclude that although few regions of the brain appear entirely immune from the untoward consequences of alcoholism, the regions most at risk include the prefrontal cortex and subjacent white matter, cerebellar sites, and white matter structures and tracts, including the corpus callosum. Subsequent sections review evidence regarding the brain consequences of excessive alcohol consumption that appear to be reversible in the first weeks and months of sobriety and those that persist even with extended sobriety. The reader is referred elsewhere for fuller descriptions of the MRI methods and, quantification approaches, as well as artifactual considerations that limit the usefulness of brain-imaging data (Adalsteinsson et al. 2002; Hennig et al. 2003; Pfefferbaum et al. 2006*b*; Rosenbloom et al. 2003).

## Studies Comparing Alcoholics and Nonalcoholics

### Structural MRI Evidence for Alcohol’s Effects on Brain Structures

MRI studies that compare patients with chronic alcoholism to people without a history of excessive alcohol use typically find smaller volumes of gray matter (Cardenas et al. 2005; Chanraud et al. 2007; Fein et al. 2002; Gazdzinski et al. 2005*b*; Jernigan et al. 1991; Pfefferbaum et al. 1992) in the cerebral cortex, the folded outer layer of the brain. Gray matter differences are more marked in alcoholics who smoke than in those who do not smoke (Gazdzinski et al. 2005*b*). The volume of white matter lying beneath and beside cortical gray matter also is smaller in alcoholics than in nonalcoholics (Chanraud et al. 2007; Gazdzinski et al. 2005*b*; Pfefferbaum et al. 1992). Older alcoholics show greater gray and white matter volume deficits relative to age-matched control subjects than younger alcoholics, especially in the frontal lobes (Cardenas et al. 2005; Pfefferbaum et al. 1997), even when older alcoholics have consumed equivalent amounts of alcohol over their lifetime as younger alcoholics.

This age–alcoholism interaction suggests that as people age, their brains become more vulnerable to the effects of excessive alcohol consumption (Pfefferbaum et al. 1992). Studies of community samples of men without histories of alcohol dependence found that heavy drinking (about four drinks a day) was associated with significantly more age-related reduction in frontal lobe volume (Kubota et al. 2001) and showed a negative association between lifetime alcohol intake and gray matter volume in the frontal lobes relative to lower-alcohol–consuming counter-parts (Taki et al. 2006).

MRI of the cerebral cortex also shows that temporal lobe white matter volume deficits are prevalent in patients with a history of alcohol withdrawal seizures (Sullivan et al. 1996). Studies show that the greatest cortical shrinkage in alcoholism without concurrent disease or other comorbidities (i.e., uncomplicated alcoholism) occurs in the frontal lobes (Pfefferbaum et al. 1997), which subserve reasoning, working memory, and problem solving (Oscar-Berman and Marinkovic 2007). These findings are consistent with postmortem studies (Courville 1955; Harper and Kril 1993).

In addition, the cerebellum, or “little brain,” which lies behind and beneath the cerebral cortex, also is adversely affected even in patients with uncomplicated alcoholism (Chanraud et al. 2007; Sullivan et al. 2000*a*). These in vivo findings are consistent with postmortem reports of shrinkage, prominent in large neurons in part of the cerebellum known as the anterior superior vermis (Harper 1998).

Traditionally, the cerebellum was thought to be mainly responsible for controlling motor behavior, including balance. Alcohol-related damage to this structure is presumed to be responsible for the truncal and lower-limb motor deficits that cause lack of coordination and are observed commonly in patients with Wernicke-Korsakoff Syndrome^4^ (Victor et al. 1971). More recent studies on the role of the cerebellum and the extensive circuits linking it to subcortical and cortical regions have highlighted its critical role for higher-order functions classically associated with the frontal lobes (Schmahmann 1997). Damage to the central portion of the cerebellum (i.e., the vermis) from excessive alcohol consumption thus contributes not only to deficits of balance and gait in chronic alcoholics (Sullivan et al. 2000*a*, 2006) but also to impairment in functions such as problem solving and working memory (Desmond et al. 1998; Sullivan et al. 2003*a*).

Structural MRI studies have shown that subcortical and brainstem structures known to be affected in severe neurological syndromes such as Marchiafava-Bignami disease,^5^ central pontine myelinolysis, alcoholic cerebellar degeneration, and Korsakoff’s syndrome, which are all associated with excessive alcohol consumption or associated nutritional deficiency, also are affected in patients with uncomplicated alcoholism, albeit to a lesser degree (Sullivan and Pfefferbaum 2009). These structures include bodies of white matter, such as the corpus callosum (Estruch et al. 1997; Hommer et al. 1996; Pfefferbaum et al. 2006*b*) and pons (Bloomer et al. 2004; Sullivan and Pfefferbaum 2001; Sullivan et al. 2005); subcortical basal ganglia structures such as the thalamus (Sullivan et al. 2003*b*), caudate, and putamen (Sullivan et al. 2005); and memory-related structures such as the mammillary bodies (Sullivan et al. 1999) and anterior hippocampus (Agartz et al. 1999; Beresford et al. 2006; Bleich et al. 2003; Sullivan et al. 1995).

### DTI Evidence for Compromised White Matter Integrity

Studies investigating the effects of alcohol on brain white matter microstructure must first account for the normal variations in the extent to which water molecules are constrained in white matter (i.e., anisotropy) (see DTI textbox and figures for examples) across brain regions depending on the linearity and homogeneity of the local fiber structure as well as normal effects of age. It now is well established that normal aging accounts for significant variation, particularly in frontal regions (for reviews see Minati et al. 2007; Pfefferbaum and Sullivan 2005; Sullivan and Pfefferbaum 2007; Wozniak and Lim 2006).

Both postmortem (Wiggins et al. 1988) and in vivo (Pfefferbaum et al. 2006*b*) studies have found age–alcoholism interactions in the macrostructure of the corpus callosum. DTI studies of corpus callosal microstructure have confirmed these observations. Pfefferbaum and colleagues have reported abnormally low anisotropy in regions of the corpus callosum as well as in a white matter region above the cerebellum (i.e., termed the centrum semiovale) in alcoholic men (Pfefferbaum et al. 2000) and women (Pfefferbaum and Sullivan 2002). The researchers identified these microstructural deficits, even though in some cases, structural MRI did not detect size deficits in the corpus callosum. A later study (Pfefferbaum et al. 2006*b*) of the corpus callosum that tested a different group of alcoholic and control men and women found that an index of white matter tissue compromise (i.e., diffusivity) was strikingly higher in alcoholic men and women than in control subjects and showed regionally nonspecific, substantial correlations with macrostructural volume. Furthermore, older alcoholics had greater abnormalities for their age in both diffusivity and fractional anisotropy (FA) (see DTI textbox) than younger alcoholics, a finding that likely reflects both the loss of the axons’ outer protective sheath (i.e., demyelination), and deletion of axons that form the center of white matter tracts.

A recent DTI study using quantitative tractography to investigate the integrity of white matter fiber bundles revealed signs of fiber tract degradation, particularly of myelin, in frontal and superior brain regions (frontal forceps, internal and external capsules, fornix, and superior cingulate and longitudinal fasciculi) of alcoholics relative to controls (Pfefferbaum et al., in press). Greater lifetime alcohol consumption by alcoholic men correlated with poorer condition of some of these fiber bundles. When matched for alcohol exposure, alcoholic women showed more DTI signs of white matter degradation than alcoholic men, suggesting that women are at enhanced risk for alcoholism-related degradation in selective white matter systems. Another DTI study reported that detoxified alcoholics had nearly 20 percent fewer fibers per unit volume of white matter in tracts between the midbrain and pons, which was predictive of poorer performance on a test of mental flexibility (Chanraud et al. 2008).

**Diffusion Tensor Imaging**

**Figure 2:**
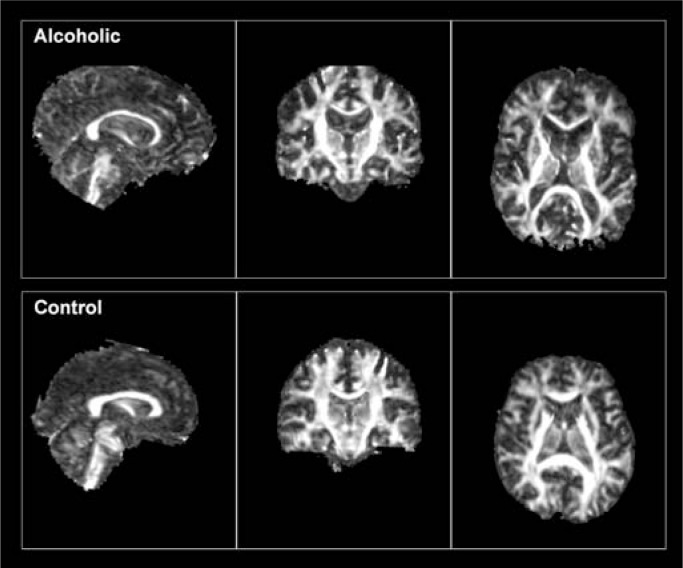
Images from sagittal (left), coronal (center), and axial (right) views of a 57-year-old alcoholic man (upper panel) and a 54-year-old control man (lower panel) displaying values for fractional anistrophy (FA) and illustrating clearly the white matter architecture of the brain. Note the more robust appearing white matter structures in the control than the alcoholic. The sagittal view highlights the corpus callosum and the pons and brain stem structures. The coronal view illustrates how the corpus callosum (above the ventricles) links left and right hemispheres. The axial view illustrates the genu and splenium of the corpus callosum.

Despite its neuroanatomical detail, conventional magnetic resonance imaging (MRI) typically does not reveal information about the microstructure of brain tissue and its components, such as axons, and myelin in white matter or cell orientation in gray matter. By contrast, diffusion tensor imaging (DTI) measures the diffusion of water molecules within brain cells and in extracellular spaces by making the following assumptions: when unconstrained by barriers such as cell membranes (and as occurs in the fluid-filled space of the lateral ventricles), water molecules move equally in all directions, and this movement is called isotropic, meaning the same in every direction. However, in tissue with a regular and orderly microstructure, such as brain white matter (Waxman et al. 1995), the water molecules are constrained by the white matter tracts to move mainly in the orientation of specific fiber lines, and this movement is called anisotropic, meaning not the same in every direction. Anisotropy is calculated within each image element, or voxel, and expressed as a fraction that reflects the extent to which water molecules are constrained in that voxel. Fractional anisotropy (FA) (Pierpaoli and Basser 1996) can range from 0 (perfect isotropy) for cerebrospinal fluid (CSF)-filled spaces to 1 (perfect anisotropy) for highly organized, parallel bands of white matter such as the corpus callosum. In contrast to FA, diffusion (diffusivity) measures the freedom of motion and generally is high in CSF, much lower in gray matter, and even lower in white matter. The axonal cytoskeleton, including myelin and the linear orientation of neurofilaments that keep anisotropy high in healthy white matter, can be perturbed with trauma or disease, including alcoholism, leading to diminished anisotropy, marking disruption of linearity (Arfanakis et al. 2002). In addition, disease-related accumulation of fluids in the extracellular spaces between fibers provides an avenue for water movement in white matter, increasing diffusivity. Thus, high FA and low diffusivity generally reflect healthy white matter. On an FA image, higher intensity signals denote higher FA and typically highlight the white matter skeleton (see figure 2) (For detailed reviews on DTI methods see Horsfield and Jones 2002; Le Bihan 2003, 2007; Mori and Zhang 2006; Pfefferbaum and Sullivan 2005).

— Margaret J. Rosenbloom, and Adolf Pfefferbaum, M.D.

ReferencesArfanakisKHaughtonVMCarewJDDiffusion tensor MR imaging in diffuse axonal injuryAmerican Journal of Neuroradiology23794802200212006280PMC7974716HorsfieldMAJonesDKApplications of diffusion-weighted and diffusion tensor MRI to white matter diseases: A reviewNMR in Biomedicine1557057720021248910310.1002/nbm.787Le BihanDLooking into the functional architecture of the brain with diffusion MRINature Reviews Neuroscience446948020031277811910.1038/nrn1119Le BihanDThe ‘wet mind’: Water and functional neuroimagingPhysics in Medicine and Biology52R57R9020071737490910.1088/0031-9155/52/7/R02MoriSZhangJPrinciples of diffusion tensor imaging and its applications to basic neuroscience researchNeuron5152753920061695015210.1016/j.neuron.2006.08.012PfefferbaumASullivanEVDiffusion MR imaging in neuropsychiatry and agingGillardJWaldmanABarkerPClinical MR Neuroimaging: Diffusion, Perfusion and SpectroscopyCambridgeCambridge University Press2005558578PierpaoliCBasserPJTowards a quantitative assessment of diffusion anisotropyMagnetic Resonance in Medicine368939061996894635510.1002/mrm.1910360612WaxmanSGKocsisJDStysPKThe Axon: Structure, Function and PathophysiologyNew YorkOxford University Press1995

### Behavioral Evidence for Deficits in Cognitive and Motor Function

Neuroimaging studies have documented a range of consequences of chronic excessive alcohol use, including volume deficits in the frontal lobes and cerebellum and compromised integrity of white matter microstructure.

What are the practical consequences of these changes in terms of deficits in cognitive and motor function? A significant percentage of recovering chronic alcoholics exhibit mild-to-moderate deficits in complex cognitive processes. Importantly, functions tend to be impaired but not completely lost. Typically, the processes affected are visuospatial abilities; psychomotor speed; executive functions, such as working memory, problem solving, temporal ordering, and response inhibition; and gait and balance (for reviews see Fein et al. 1990; Moselhy et al. 2001; Nixon et al. 2002; Oscar-Berman 2000; Oscar-Berman and Marinkovic 2007; Sullivan 2000), evidenced in both alcoholic women (Sullivan et al. 2002*b*) and men (Sullivan et al. 2000*c*).

Despite the multiplicity of behavioral deficits associated with chronic alcohol dependence, only a few studies have been able to demonstrate links between relatively specific component processes and measures of localized volume deficit in particular rather than broadly defined brain regions (e.g., Chanraud et al. 2007). By contrast, sensory or motor functions that draw on focal rather than multiple brain regions for successful performance have been more readily associated statistically with the relevant brain region. For example, olfactory discrimination ability is correlated with thalamic volumes (Shear et al. 1992), performance on a simulated gambling task is associated with atrophy of the amygdala (Fein et al. 2006), and postural stability is correlated selectively with anterior superior cerebellar vermian volumes (e.g., Sullivan et al. 2000*a*, 2006).

The difficulty in finding simple associations between alcohol-related deficits in specific brain structures and specific cognitive functions has led to the hypothesis that the mechanism underlying alcohol-related cognitive compromise may arise from the degradation of selective neural circuitry connecting cortical sites rather than either specific damage at the site or complete disconnection of white matter tracts connecting the cortical sites (Sullivan and Pfefferbaum 2005).

In this context, DTI evidence for reduced integrity of white matter structures is particularly relevant. Several recent studies have shown that performance on tests of different cognitive processes such as attention, working memory, or visuospatial ability are each selectively related to microstructural integrity of different regions of the corpus callosum in alcoholics. In one study, Pfefferbaum and colleagues (2006*b*) calculated a composite score for working memory, classically considered a “frontal lobe” function, based on Backward Digit Span and Block Spans from the Wechsler Memory Scale–Revised (Wechsler 1987) and Trail Making Part B (Lezak 1995), and also assessed visuospatial ability with the Matrix Reasoning Subtest of the Wechsler Abbreviated Scale of Intelligence (Wechsler 1999), performance on which is selectively impaired by lesions of the parietal cortex (Villa et al. 1990). A series of analyses identified a double dissociation in alcoholics—that is, low scores on the working memory composite correlated with high diffusivity in the genu but not the splenium of the corpus callosum, whereas low scores on matrix reasoning correlated with high diffusivity in the splenium but not the genu of the corpus callosum.

**Figure 3:**
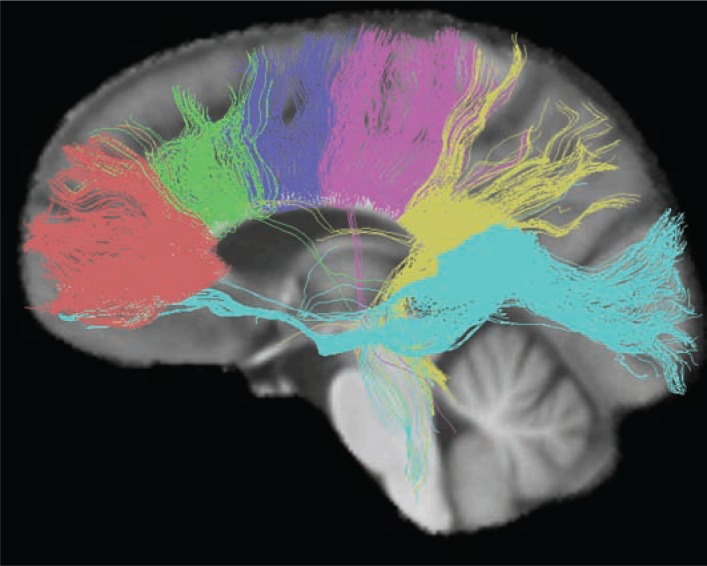
Fiber tracts from six segments of the corpus callosum providing inter-hemispheric linkage between specific cortical regions. The six segments and their fibers are identified as genu (coral), premotor (green), sensory-motor (purple), parietal (pink), temporal (yellow), and splenium (blue).

In another study (Rosenbloom et al. 2008), tractography measure of fibers connecting the left and right hemispheres through the genu of the corpus callosum selectively predicted performance on the Digit Symbol Test (see textbox and figure 3 for explanation of tractography). The associations observed are consistent with the topographically compartmentalized tracts of the corpus callosum in which the genu connects lateralized frontal sites and the splenium connects lateralized parietal and occipital sites (de Lacoste et al. 1985; Pandya and Seltzer 1986).

### fMRI Evidence That the Brain Compensates for Cognitive Deficits

fMRI is used to detect which brain systems are invoked while performing an experimental task and how alcoholics and control participants may differ in the systems activated to perform a common task. Such studies have assessed working memory (Desmond et al. 2003; Pfefferbaum et al. 2001; Tapert et al. 2001), long-term memory (Akine et al. 2007), and the ability to overcome interference from a previously learned task, in this case, color matching (De Rosa et al. 2004).

A common finding across all of these studies is that alcoholics achieve normal levels of performance but accomplish this by activating brain regions that are different from controls. This suggests that alcoholics’ brains undergo compensatory reorganization to enable them to perform at nonimpaired levels. In one study (Pfefferbaum et al. 2001), alcoholics showed less activation in prefrontal regions than control participants and more activation in posterior and inferior regions to perform a spatial working memory task. In another study (Desmond et al. 2003), alcoholics showed greater activations in the left prefrontal cortex compared with controls and also activated the right superior cerebellum, not invoked by controls, to perform a verbal working memory task on par with control subjects, suggesting that the cerebellum augmented or compensated for functional impairment of the prefrontal cortex in alcoholics. In a study of cognitive interference, in which respondents first learned to make one kind of response to a specific stimulus type and then had to unlearn it and apply a new response (De Rosa et al. 2004), alcoholics recruited higher-order, frontal neural systems to perform a task that usually was performed automatically by the control participants, who activated lower-level, subcortical systems to carry out the same task.

**Tractography**

New advances in image processing now enable researchers to characterize the integrity of specific white matter structures, such as the corpus callosum and broad cortical regions of white matter, view white matter fiber systems (Lehericy et al. 2004; Mori et al. 2005; Stieltjes et al. 2001; Xu et al. 2002), and quantify fractional anisotropy (FA) and diffusivity along the length of identified fiber bundles (Gerig et al. 2005; Sullivan et al. 2006). This approach, referred to as quantitative fiber tracking, does not actually identify anatomically specific fibers or fiber bundles as detected histologically. Rather, it is a statistical representation of the voxel-to-voxel coherence of DTI-detectable water diffusion in white matter that is nonetheless increasingly being shown to represent the underlying anatomy (Schmahmann et al. 2007).

— *Margaret J. Rosenbloom, and Adolf Pfefferbaum, M.D*.

ReferencesGerigGCorougeIVachetCQuantitative analysis of diffusion properties of white matter fiber tracts: a validation study (Abstract)13th Proceedings of the International Society for Magnetic Resonance in MedicineMiami, FL2005(Abstract no. 1337)LehericySDucrosMVan De MoortelePFDiffusion tensor fiber tracking shows distinct corticostriatal circuits in humansAnnals of Neurology5552252920041504889110.1002/ana.20030MoriSWakanaSNagae-PoetscherLMVan ZijlPMCAn Atlas of Human White MatterAmsterdam, The NetherlandsElsevierr B.V.2005SchmahmannJDPandyaDNWangRAssociation fibre pathways of the brain: Parallel observations from diffusion spectrum imaging and autoradiographyBrain13063065320071729336110.1093/brain/awl359StieltjesBKaufmannWEVan ZijlPCDiffusion tensor imaging and axonal tracking in the human brainstemNeuroImage1472373520011150654410.1006/nimg.2001.0861SullivanEVAdalsteinssonEPfefferbaumASelective age-related degradation of anterior callosal fiber bundles quantified in vivo with fiber trackingCerebral Cortex161030103920061620793210.1093/cercor/bhj045XuDMoriSSolaiyappanMA framework for callosal fiber distribution analysisNeuroImage171131114320021241425510.1006/nimg.2002.1285

fMRI studies of alcoholics suggest the importance of cerebellar activation in functions that otherwise would be frontal lobe tasks and the activation of frontal regions in functions that otherwise would be lower-level tasks. One interpretation is that this additional activation enables alcoholics to achieve normal levels of coordinated performance despite evidence for cerebellar dysmorphology but at a cost to processing capacity. This functional style observed in alcoholics, which may be compensatory, has been characterized as “processing inefficiency” (Nixon and Parsons 1991). Processing inefficiency traditionally is associated with conditions in which speed is sacrificed for accuracy (Nixon 1993). Alcoholics move more slowly to attain normal accuracy, as observed in a quantified version of the finger-to-nose test, which is sensitive to cerebellar functioning and in which alcoholics achieved equivalent or even smaller trajectory deviations than control subjects (Sullivan et al. 2002*a*). This performance is symptomatic of cerebellar hemisphere dysfunction, characterized by deliberation of otherwise automatic movements. When automatic processing becomes effortful, it calls on limited processing capacity, which is then unavailable for other tasks. Taken together, these phenomena suggest a common neuropsychological mechanism—processing inefficiency— and perhaps a neural mechanism— degraded white matter microstructure— as underlying these possible instances of impaired neural transmission and avenues to circumvent brain functional and structural impairment (c.f., Sullivan and Pfefferbaum 2005).

## Long-term Studies: Prospects for Recovery With Abstinence

The dynamic course of change in the “incomplete lesion” that characterizes alcoholism makes it a special model for human neuroscience study. Certain brain structural abnormalities are at least partially reversible with abstinence, perhaps through remyelination, creation of new neurons (i.e., neurogenesis), or simple cellular revoluming, and are accompanied by improvement in cognitive, sensory, and motor functions. Indeed, more than 20 years ago, Carlen and colleagues (1986) used computerized tomography (CT), an X-ray–based brain-imaging technique to demonstrate that the negative consequences of chronic excessive alcohol use on the brain are mitigated to some extent by maintaining sobriety.

More recent longitudinal MRI studies of alcoholics during short-term treatment–related abstinence, followed by continued abstinence or relapse after discharge, have found that with short-term (about 1 month) abstinence from alcohol, cortical gray matter (Pfefferbaum et al. 1995), overall brain tissue (Bartsch et al. 2007; Gazdzinski et al. 2005*a*), or hippocampal structures (Gazdzinski et al. 2008*b*) increase in volume. After discharge, those who maintain sobriety show reduced volume of the third ventricle (Pfefferbaum et al. 1995) or a general increase in brain volume (Gazdzinski et al. 2005*a*) that favors frontal and temporal lobes (Cardenas et al. 2007), whereas those who relapse show expansion of the third ventricle and shrinkage of white matter (Pfefferbaum et al. 1995) or loss of overall brain tissue relative to that seen at study entry (Cardenas et al. 2007; Gazdzinski et al. 2005*a*) (see figure 5). Additional studies have highlighted that cortical white matter volume may be particularly amenable to recovery with abstinence (Agartz et al. 2003; Meyerhoff 2005; O’Neill et al. 2001; Shear et al. 1994) or vulnerable to further decline with continued drinking (Pfefferbaum et al. 1995).

Animal studies have revealed neurogenesis in the hippocampus in long-abstinent animals (Nixon and Crews 2004). Although no equivalent evidence currently is available in humans, one longitudinal MRI study (Cardenas et al. 2007) reported increased temporal lobe volume and MR spectroscopic^6^ studies have shown improved neuronal integrity in abstinent alcoholics (Durazzo et al. 2006).

In studies of extended sobriety (i.e., 5 years), research focuses on comparing those who maintained sobriety with those who resumed drinking (Muuronen et al. 1989; Pfefferbaum et al. 1998). Long-term prolonged sobriety was associated with improvement or stabilization of measures of brain tissue volume, whereas return to drinking was associated with increased ventricular volume. In addition, among those who continued drinking, cortical gray matter loss over the follow-up period, especially in the frontal lobes, was associated with the degree of excessive drinking in retested alcoholics (Pfefferbaum et al. 1998). Several factors may diminish the likelihood of recovery of brain structure with sobriety—such as older age, heavier alcohol consumption, concurrent hepatic disease, history of withdrawal seizures, malnutrition, and concurrent smoking. Unfortunately, few studies to date have obtained longitudinal data on large enough samples to model these factors effectively, although one study (Yeh et al. 2007) demonstrated that greater smoking and drinking severity before abstinence was associated with greater reduction of ventricular volume during abstinence. Investigators have limited control over whether participants in longitudinal studies maintain abstinence or continue drinking. By contrast, studies of animals, reviewed below, give researchers control over the outcomes of abstinence and relapse.

### Abstinence and Cognitive Improvements in Humans

A growing number of longitudinal neuropsychological studies report significantly better scores on tests of working memory, visuospatial abilities, and gait and balance with abstinence from alcohol. Some components of these functional domains recover faster (Rosenbloom et al. 2004) or more fully than others (e.g., Becker et al. 1983; Brandt et al. 1983; Mann et al. 1999; Nixon and Glenn 1995; Parsons et al. 1987; Sullivan et al. 2000*b*), but at least a measurable degree of recovery typically accompanies prolonged sobriety, suggesting that the changes observed with neuroimaging have functional consequences.

**Functional Magnetic Resonance Imaging**

**Figure 4:**
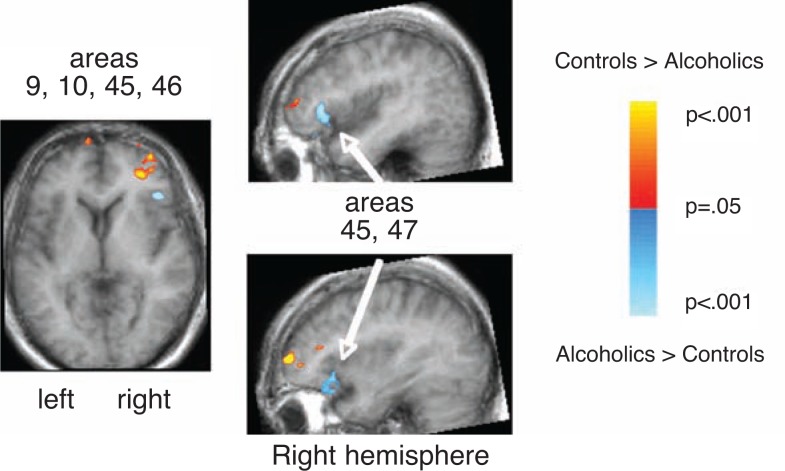
The results of a functional magnetic resonance imaging (fMRI) study in which alcoholics and control subjects performed a spatial location task while lying in the MR scanner. Three views of the brain illustrate the regions where alcoholics showed more (blue) or less (red) activation than control subjects when judging whether a dot on a slide was in the center, compared with a rest period (Pfefferbaum et al. 2001). The control subjects showed more activation in prefrontal areas (Brodmann’s areas 9, 10, 45, and 46), whereas the alcoholics showed more activation in inferior and posterior frontal locations (Brodmann’s areas 45 and 47) in the right hemisphere. SOURCE: Pfefferbaum, A.; Desmond, J.E.; Galloway, C.; et al. Reorganization of frontal systems used by alcoholics for spatial working memory: An fMRI study. *NeuroImage* 14:7–20, 2001. PMID: 11525339

**Figure 5:**
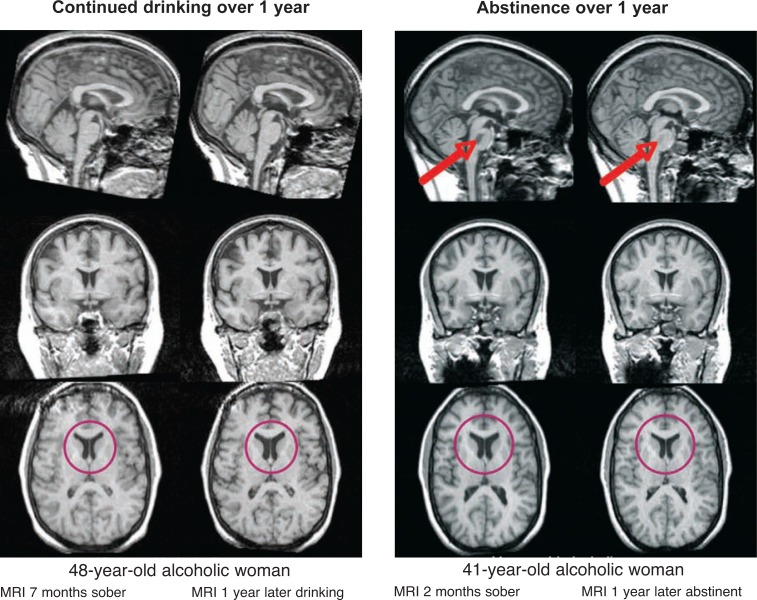
Effect of abstinence. Brain images show the contrast between an alcoholic who continues to drink and one who maintains sobriety. For both cases, the images to the left were obtained after a period of sobriety and the images to the right were obtained 1 year later. In the lower panel for each woman, we see expansion of the lateral ventricles with continued drinking and reduction of the lateral ventricles with continued sobriety. In the upper panels we see that a lesion in the pons, clearly visible in the first image, has resolved after a year of sobriety.

The magnetic resonance imaging (MRI) and diffusion tensor imaging (DTI) techniques described above each provide a static representation of the brain. By contrast, functional MRI (fMRI) exploits the MRI-visible signal contrast between oxygenated (higher signal) and deoxygenated (lower signal) hemoglobin as it flows through small blood vessels in given brain regions. Neural activity while performing a cognitive, motor, or sensory task increases the ratio of oxygenated to deoxygenated hemoglobin in the blood of neighboring vasculture and enhances the MR signal. This blood oxygen level– dependent (BOLD) contrast mechanism does not directly measure blood flow or neuronal activity but rather the small, rapid changes in the blood’s paramagnetic properties (related to unpaired electrons in the blood) that can be imaged by rapid sampling over the spatial domain (Logothetis and Pfeuffer 2004). Changes in levels of oxygenated hemoglobin in blood vessels, the hemodynamic response that occurs in response to experimental manipulations, the affect local homogeneity of an MR signal. The BOLD effect is localized by measuring the difference between oxygenation at the time a specific task is completed and at a rest period or another (control) task. The regions of the brain showing the greatest difference between active and contrast conditions are believed to be those most involved in performing the operation under investigation (Hennig et al. 2003; Toma and Nakai 2002). Contrasts between groups (e.g., alcoholics and controls) further illustrate regions of the brain where one group shows more activation while performing a specific task than the other (figure 4). Further technical details about fMRI can be found in specialized reviews (Adalsteinsson et al. 2002; Buckner and Logan 2001; Buxton 2002; Friston 2005).

— *Margaret J. Rosenbloom, and Adolf Pfefferbaum, M.D.*

ReferencesAdalsteinssonESullivanEVPfefferbaumABiochemical, functional and microstructural magnetic resonance imaging (MRI)LiuYLovingerDMMethods in Alcohol-Related Neuroscience ResearchBoca Raton, FLCRC Press2002345372BucknerRLLoganJMFunctional neuroimaging methods: PET and fMRICabezaRKingstoneAHandbook of Functional Neuroimaging of CognitionCambridge, MAMIT Press20012748BuxtonRBIntroduction to Functional Magnetic Resonance Imaging: Principles & TechniquesCambridge, UKCambridge University Press2002FristonKJModels of brain function in neuroimagingAnnual Review of Psychology56578720051570992910.1146/annurev.psych.56.091103.070311HennigJSpeckOKochMAWeillerCFunctional magnetic resonance imaging: A review of methodological aspects and clinical applicationsJournal of Magnetic Resonance Imaging1811520031281563410.1002/jmri.10330LogothetisNKPfeufferJOn the nature of the BOLD fMRI contrast mechanismMagnetic Resonance Imaging221517153120041570780110.1016/j.mri.2004.10.018TomaKNakaiTFunctional MRI in human motor control studies and clinical applicationsMagnetic Resonance in Medical Science110912020021608213210.2463/mrms.1.109

Several studies have demonstrated that improvements in brain structure may be associated with cognitive improvements. In one study, the short-term recovery of hippocampal volume over the first month of abstinence was associated with improved visuospatial memory but only in non-smoking alcoholics (Gazdzinski et al. 2008*b*). In another study, 15 alcoholics and 26 control subjects were followed for 2 years. Of the alcoholics, 10 maintained sobriety, where-as 5 relapsed to heavy drinking.

The abstainers showed improvement in general memory relative to the control participants, a behavioral change that was associated with reduced volume of the lateral ventricle. The abstainers also showed improvement in balance, which was associated with reduced volume of the fourth ventricle. The lateral and fourth ventricle are each adjacent to brain structures associated with either memory or balance, suggesting that structural brain changes could have contributed to the improved memory and balance seen in these 2-year abstinent alcoholics (Rosenbloom et al. 2007). These findings are similar to those reported in an earlier study (Sullivan et al. 2000*b*) in which participants were followedup after 2 to 12 months. Shrinkage in third-ventricle volume across all participants significantly correlated with improvement in nonverbal short-term memory. Researchers found additional relationships between brain structure and function, most involving short-term memory, among alcoholic men who had maintained complete abstinence, were light relapsers for at least 3 months, or had consumed no more than 10 drinks prior to follow-up testing.

Although there is substantial evidence now for restoration of alcohol-impaired brain structure and function with sobriety, investigators do not yet know the mechanism for either loss of brain tissue volume with drinking or its restoration with abstinence (Harper and Kril 1990). Changes in both myelination and axonal integrity in white matter and changes in the cells making up cortical gray matter are probably involved.

## Translational Studies Using In Vivo Neuroimaging in Animal Models

Many of the complexities of studying the effects of chronic excessive alcohol consumption on the brain in humans can be controlled, to some extent, by studying laboratory animals that model human alcoholism using in vivo neuroimaging techniques. To model human alcoholism, animals should at a minimum consume large amounts of alcohol, either voluntarily or by experimental exposure, and develop tolerance and withdrawal symptoms.

The rat provides a particularly useful animal model for neuroimaging studies of alcoholism because it is a suitable size for in vivo imaging (Pfefferbaum et al. 2006*a*) and because researchers can control its genetic predisposition for drinking (Li et al. 1979), nutritional status (Pfefferbaum et al. 2007), and alcohol dose and pattern (intermittent binge drinking versus regular heavy drinking) (Pfefferbaum et al. 2008) and timing of alcohol exposure during the life cycle. Furthermore, neuroimaging can be performed repeatedly over the animal’s relatively brief life cycle to measure the effects of different alcohol-dosing regimens (intermittent binge drinking versus continuous heavy drinking), nutritional manipulations, and exposure at different life stages (adolescence, adulthood, and old age).

**Figure 6:**
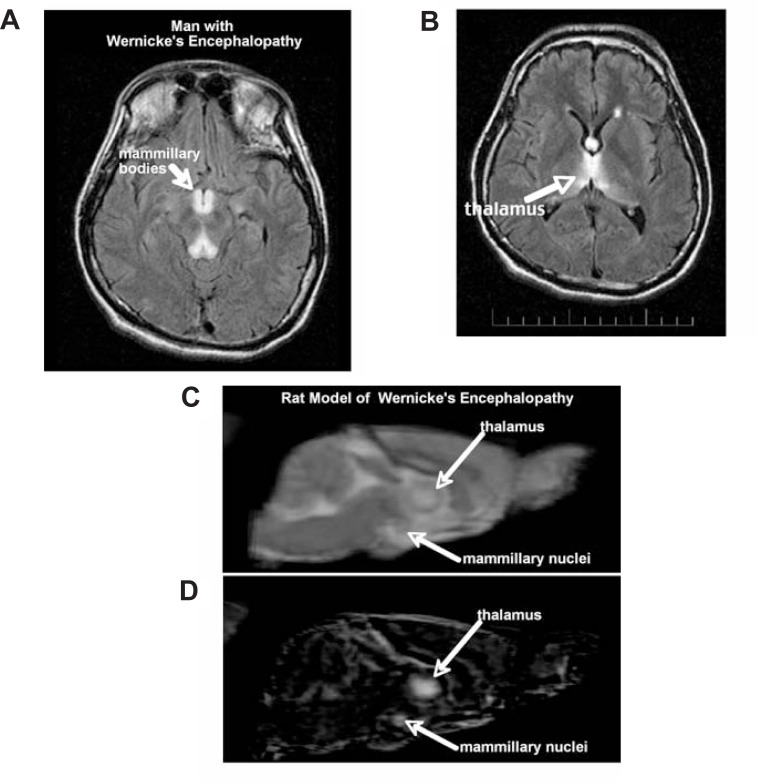
Axial MR fluid attenuated inversion recovery (FLAIR) (a subtype of MRI) image of a 35-year-old man with an acute nutritional deficiency-induced Wernicke’s encephalopathy (WE). Prominent are the hyperintense signals in the mammillary bodies (A) and thalamus (B) indicating tissue pathology. Sagittal slice of a structural image of an individual rat that has been treated with pyrithiamine to model the acute thiamine deficiency of WE (C) and a difference image—created by subtracting the mean image acquired from all rats before treatment from the mean image acquired after treatment—that highlights changes induced by pyrithiamine in pyrithiamine-treated rats (D) (taken from Pfefferbaum et al. 2007). Note the hyperintense areas in the thalamus and mammillary nuclei in the rat images that are comparable to similar areas of hyperintensity in the man with acute WE.

A 1-year study of genetically selected alcohol-preferring rats, which voluntarily drank large amounts of alcohol, found sustained ventricular enlargement and stunting of corpus callosum growth, possibly modeling the human condition (Pfefferbaum et al. 2006*a*). Following several bouts of voluntary drinking, some rats underwent thiamine depletion followed by repletion. Rats with a history of alcohol exposure plus thiamine deficiency were especially prone to developing brain lesions identified with alcoholic Wernicke’s encephalopathy (Victor et al. 1989) (see figure 6). Some of the lesions resolved following dietary improvement (Pfefferbaum et al. 2007), suggesting that, at least in rats bred to prefer alcohol, nutritional deficiency must be combined with alcohol to produce severe neurodegeneration. In a study with wild-type rats, alcohol was administered by inhalation. Animals were maintained on a good diet and did not suffer withdrawal seizures; however, they developed significant ventricular enlargement (Pfefferbaum et al. 2008), suggesting that among animals who are not selected for alcohol preference, alcohol alone can be neurotoxic. (Additional information regarding translational studies using animal models can be found in the article by Zahr and Sullivan in “Neuroscience: Part I”) (Zahr and Sullivan 2008).

## Conclusion

Studies using in vivo MR imaging have clearly documented that chronic excessive alcohol consumption leads to brain pathology. Some of this pathology, such as white matter volume reduction, is reversible with abstinence, but some appear to be enduring. Research also has demonstrated the functional consequences of the pathology. Structural imaging studies have shown correlations between brain morphology and quantitative neuropsychological testing, and functional imaging studies provide evidence for compensation of cognitive deficits.

The myriad associated and difficult-to-control concomitants of alcoholism (e.g., malnutrition, hepatic disease, head trauma, heavy smoking, and lack of exercise), the antecedents (e.g., premorbid reserve and capacity and genetic vulnerability or susceptibility), and the consumption patterns (e.g., age at onset, history of withdrawals, quantity and frequency of consumption) all may influence the observed brain changes associated with alcoholism. As such, they present unique challenges and opportunities to understand the underlying mechanism of alcoholism-induced neuropathology. Given the evidence for structural and functional repair and recovery in sober alcoholics, at least a portion of the neuropathology must be transient. This transience can be followed rigorously with animal models over time and may account for difficulties in finding specific relationships between brain structure, volume, and function in alcoholics but should provide hope for the sober alcoholic. Nonetheless, the dynamic course of alcoholism presents an important and challenging neuroscience model for understanding mechanisms of neurodegeneration, functional recovery, compensation, and processing limitations that should be applicable to any neurological condition characterized by a fluctuating course.

## Figures and Tables

**Figure 1:**
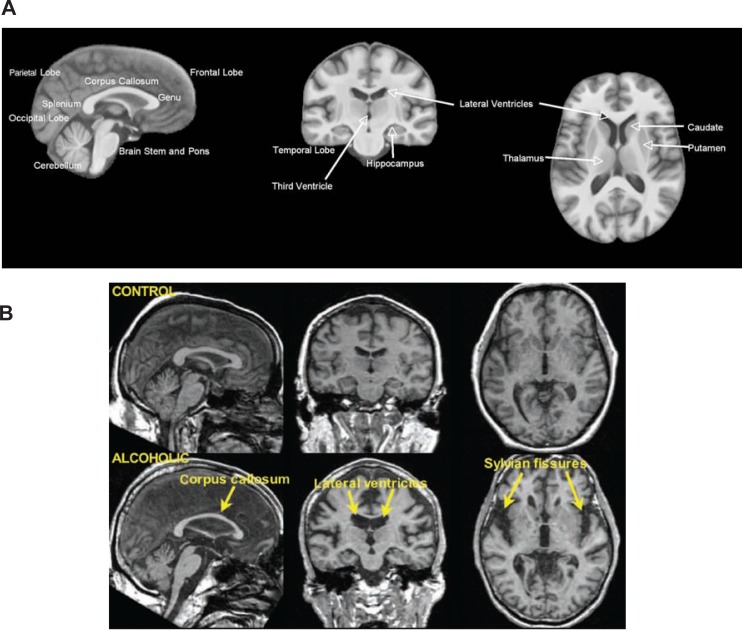
**A)** Standardized magnetic resonance imaging (MRI) of the brain viewed from the side, sagittal (left); back, coronal (middle); and above, axial (right). The dark areas represent fluid, white represents white matter, and shades of gray represent different gray matter areas and structures. Specific cortical regions and subcortical structures are labeled. **B)** MRI scans from a 53-year-old control man (upper) and a 53-year-old alcoholic man (lower) from the same views as shown above. Note the enlargement of the lateral ventricles and sulci, reduced cortical tissue, and skinnier corpus callosum in the alcoholic compared with the control.

## Footnotes

2 See figure 1, pp. 363, and textbox 1, pp. 364 for a description of MRI and examples of MRI images.

3 See the textbox on page 366 for an explanation of DTI.

4 Korsakoff’s syndrome is a memory disorder caused by thiamine deficiency.

5 Marchiafava-Bignami disease is a progressive neurological disease characterized by degeneration of the corpus callosum.

6 MR spectroscopy provides biochemical information about tissues, whereas MRI only provides structural information.
